# Depression in Employees in Privately Owned Enterprises in China: Is It Related to Work Environment and Work Ability?

**DOI:** 10.3390/ijerph10041152

**Published:** 2013-03-25

**Authors:** Jing Sun, Nicholas Buys, Xinchao Wang

**Affiliations:** 1 School of Public Health and Griffith Health Institute, Griffith University, Gold Coast campus, Parkland, Gold Coast, Q4222, Australia; 2 School of Human Services and Social Work, and Griffith Health Institute, Griffith University, Gold Coast campus, Parkland, Gold Coast, Q4222, Australia; E-Mail: n.buys@griffith.edu.au; 3 Guanghua School of Management, Peking University, Beijing 100871, China; E-Mail: xcwang@gsm.pku.edu.cn

**Keywords:** work place depression, work environment, work ability, privately owned enterprise

## Abstract

This study examines the individual and workplace factors related to depression and stress in a large privately owned enterprise in China. The cross-sectional study design involved 13 privately owned retail enterprises in China. A self-report survey was administered to 4,847 employees aged 18–54 recruited through the management boards of the 13 enterprises. A chi-square test was used to compare differences between the depressed and non-depressed groups on a number of demographic variables and chronic diseases. Logistic regression analysis was performed to assess depression in relation to individual factors (work ability and resilience) and organisational environmental factors (workplace ethos and culture, psychosocial environment and health promotion policies and activities). Significant relationships were found between employee depression all personal factors, and one organisational environmental factor. Personal factors include poor work ability and low resilience, while workplace factors include workplace ethos and culture. The primary organisational environmental factor was a low level of enterprise ethos and culture.

## 1. Introduction

Depression in the workplace is a global concern, including in China [1]. Psychological distress is often expressed as depression and anxiety, which are the most common mental illnesses in the workplace [2]. According to one cross-national study conducted in China [3], the prevalence of major depression disorders was 12 per cent in 2011. An investigation of work-related factors that may be involved is warranted by the fact that the prevalence of depression is higher in China than in other Asian countries [3].

Depression in the workplace is considered to be related to a number of personal and organisational level factors. At a personal level, an employee’s ability to cope with and meet the physical and psychological demands of his or her job is predictive of depression [1,2,4]. Karasek and Theorell [4] proposed a demand-control social support model in which high job demand and low control, are seen as most likely to cause stress and health problems. According to this model , “high-strain jobs” (the most risky type of job) are characterised by high psychological demands, low control, lack of resources and social support, resulting in fatigue, anxiety and depression among workers [4]. On an organisational level studies from a culture-work-health perspective indicate the organisational environment may be the primary cause of distress [5]. The essence of organisational ethos and culture is in the underlying assumptions, values and beliefs that have been jointly learned and taken for granted by all levels of employees from the shop floor to management. The Culture-Work-Health Model proposes that factors, such as an organisation’s management system, structure, and social environment, are important to workers’ mental health [5]. The organizational ethos and culture also conveys to employees expectations about interrelationships between themselves and managers and co-workers in terms of behaviour and communication [5]. It is argued that variables relating to the organisational ethos and culture that are positively embedded in the workplace can protect against stress and depression through modifying, ameliorating or altering a person’s response to the negative effects of risk factors [6].

At an organisational level, depression has been found to be negatively related to a number of workplace psychosocial and environmental factors, including a safe and supportive environment, positive relationships with colleagues and supervisors, and opportunities for success [7]. Evidence suggests that supportive environments are strongly associated with an organisational focus on physical and mental health and improved productivity [8]. Such variables may have a decisive impact on an individual’s ability to cope with depression. They are crucial in determining the extent to which an employee can cope with workplace challenges, environmental changes and conflicts with managers and co-workers in the workplace [9]. Organisational environmental factors of importance in the workplace also include the availability of health promotion programs and health promotion activities, the expectations and work standards from employers, and opportunities for positive relationships with one’s supervisor in the workplace [8]. It is evident from the literature that a better understanding of how to promote and manage mental health problems can help enterprises to save costs and facilitate a more engaged and productive workforce [10,11].

Chronic health conditions in the workplace such as hypertension, cardiovascular disease and arthritis also frequently contribute to depression [12,13]. Such conditions are often exacerbated by the lack of appropriate health policies and health promotion activities to improve health in the workplace [11,14,15]. It is therefore important that workplace environments having health policy to encourage healthy behaviours in areas such as exercise and nutrition, and implement strategies for resolution of conflicts between employees, co-workers and managers. In summary, research has shown that work-related factors may contribute much to depression [16] as personal level factors.

Despite the adverse impact of depression on work productivity in enterprises, little research has been conducted on this issue in China. The purpose of this study is therefore to examine whether an individual’s ability to cope with work demands and the organisational environment are associated with depression in employees in Chinese enterprises. It is hypothesised that both personal factors (low working ability and work ability to meet physical and psychological demands, low level of resilience), and organisational environmental factors (availability of health promotion programs, poor level of ethos and culture, social environment, and enterprise health policy) are significantly related to the likelihood of having depression.

## 2. Methods

### 2.1. Sampling and Procedure

The Credibility Retail Enterprise is one of the largest and fastest-growing retail companies in China. It has been frequently cited in economic and development research as a typical example of a contemporary Chinese retail enterprise development. The company was established in Chang Zhou city in 1984 with only 30 employees and has now expanded to 13 companies with over 20,000 employees located in 13 rural, semi-rural and capital cities across He Bei and Shan Dong provinces. The Credibility Retail Enterprise sells a range of well-known high quality products including basic commodities and Chinese nationally branded goods, such as Haier Fridges and Li Ning sports products. The company was selected for the current study after its company leaders’ approached Peking University to assist in addressing their concerns about employees’ mental health problems. The thirteen enterprises were subsequently approached through their management boards to participate in the study. Data were collected from July 2009 by means of a survey from a randomly selected sample of 400 employees from each enterprise. A total of 5,200 employees were invited to take part in the study, with 4,847 people completing the survey, a high response rate of 93.2 per cent. Working age employees aged 18 to 55 were invited to participate in the study. The company funded all the services provided in the interventions. Ethical clearance for the study was obtained from the Research Committee at Peking University and written informed consent was obtained from participants.

### 2.2. Measures

Socio-demographic information collected included age, sex, place of origin, marital status, family income, education and occupation. Self-reported questionnaires, that were simplified Chinese versions of the original scales, were included in the survey. This included the General Health Questionnaire, Work Ability Questionnaire, Resilience and Enterprise Environment Questionnaire. 

The self-reporting General Health Questionnaire (GHQ-30) [17] was chosen for this study as it has been validated for use in similar studies that have assessed depression in workplace employees [18]. The Chinese version of the GHQ-30 has also been validated in the Chinese population [19] and has a high level of internal consistency [19], with a Cronbach alpha of 0.85 for the general population and 0.9 for the population with mental health issues. Respondents were asked to choose from four possible responses in a Likert format, where 0 is “rarely or none of the time”, and 3 is “almost or all of the time (5–7 days)”. Scores range from 0 to 30, with higher scores reflecting greater levels of depression.

Clinical interviews, conducted by qualified psychiatrists, were used to diagnose depression in this study to randomly selected 300 employees who reported to have high GHQ-30 scores more than 14. The clinical interview was based on the Diagnostic and Statistical Manual of Mental Diseases Fourth edition (DSM-IV) [20]. As a result of these interviews, 262 employees were diagnosed by doctors to have clinical level depression.

**Figure 1:**
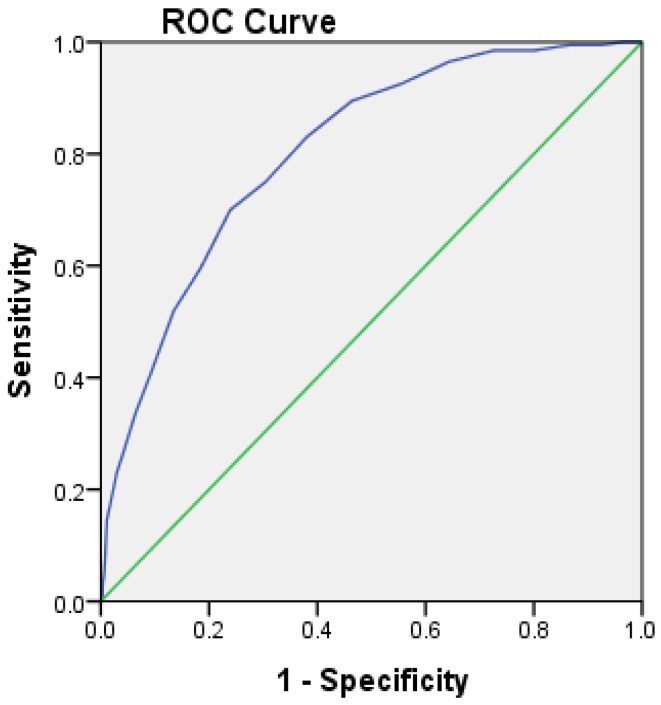
Sensitivity and specificity test of general health questionnaire (GHQ)-30 using ROC curve.

To validate GHQ-30 and provide sensitive cut-off score for the classification of people into depression and non-depression groups for this study, a sensitivity and specificity test using the ROC curve provided a cut-off score of GHQ-30 against clinical interview diagnosis results. A cut-off score more than 10.5 is suggestive of the presence of depression measured by GHQ, as it is strongly associated with clinically diagnosed major depression. This was verified by good level of sensitivity (0.75) and specificity (0.73) analyses in the current sample and the area under the ROC curve was 0.82 for depression (See Figure 1), indicating the GHQ-30 is a sensitive tool to detect distressed employees. Positive predictive value is 0.25 in this study. This validation method has been reported in a similar study and was found to be robust [2]. Regardless, the term depression should be interpreted cautiously in this study as short term depressive mood and stress, as it remains unknown which employees fulfilled the Diagnostic and Statistical Manual of Mental Disorders or other diagnostic criteria for major depression. For the ease of data analysis and reporting purpose, the employees with GHQ-30 less than 10.5 will be classified to be in normal group, and those with GHQ-30 scores of 10.5 and more to be in the depression group.

The ability of individuals to cope with physical and psychological work demands were measured by two items selected from the Work Ability Index [21]: “How do you rate your current work ability with respect to the physical demands of your work?” and “How do you rate your current work ability with respect to the psychological demands of your work?”. A five point Likert Scale was used with responses ranging from “very poor” (coded as one) to “very good” (coded as five). The reliability analysis showed the questionnaire had moderate level of reliability, with a Cronbach alpha of 0.55 for the current study.

The resilience relating to ability to cope with stress and recover from stressful situations was measured by the five-item Brief Resilience Scale [22]. The scale has a good level of reliability, with a Cronbach alpha of 0.73 for the current study.

The Enterprise Environment Questionnaire, derived from the University Organizational Health Questionnaire [23], consists of 28 items, modified to reflect the employees’ perception of the enterprise environment. The organisational environment variables examined included employees’ perceptions of the availability of health promotion activities, the enterprise ethos and culture, the extent to which a supportive psychosocial environment is present, and enterprise health policy. The Questionnaire uses a five point Likert Scale, with responses ranging from “strongly disagree” (coded as one) to “strongly agree” (coded as five). A high score on this questionnaire and subscales indicates a high level of perception by employees of a positive organisational environment and support system. Factor analysis demonstrated that the scree plot of eigenvalues indicated that there were four factors—one main factor and three smaller factors—explaining approximately 31.72%, 9.80%, 5.54% and 4.16% of the variances in enterprise environment questionnaire respectively, with total variances 51.22% explained by the four factor. The four factors are: availability of health promotion programs, enterprise ethos and culture, enterprise social environment, and enterprise health policy. (1) Availability of health promotion programs include questions related to training program provisions to promote mental health, social and emotional wellbeing, counselling services to help with mental health problems, workshop to train employees decision making and assertiveness. (2) Enterprise ethos and culture factors, include items relating to opportunity to encourage employees’ success, appropriate level of performance expectations, respect and recognition of employees’ achievement, are also significantly related to less probability to have depression. (3) The social environment includes items related to good relationship between employees and line managers. (4) Enterprise health policy includes the development of clear policies and regulations by the enterprise to ban smoking, alcohol consumption and drug use and to promote mental health and social and emotional wellbeing. The reliability analysis for the current study showed the questionnaire had high level of reliability and validity, with a Cronbach alpha of 0.91. Fifty-one percent of the variance was accounted for by the 28 items, indicating that a substantial proportion of organisational environment issues were explained by the questionnaire.

To exclude the confounding effect of chronic disease on the relationship between depression and personal and organisational environmental factors, information regarding chronic disease conditions including injury, diabetes, type 2 diabetes, heart disease, hypertension, cancer, asthma, anxiety, schizophrenia, psychosis, eating disorders, and other diseases, was also collected. The chronic conditions were diagnosed by physicians and if employees have one of the chronic conditions, they were classified to be in chronic disease group. People who did not have any of the chronic conditions were classified into the normal and healthy group.

### 2.3. Data Analysis

The prevalence of depression in the 13 enterprises was calculated according to the GHQ cut-off point of 10.5. A logistical regression model was developed to identify factors independently related to current depression in employees. Owing to the large number of inter-correlated predictor variables, a multivariate model was constructed to identify the most salient risk factors and the most parsimonious model for predicting the depression. Confounding factors that were not significant predictors in the bivariate analysis for demographic variables and chronic diseases were not included in the final multiple logistic regression model (see Table 1, Table 2). Following that, stepwise logistic regression model was used to assess the importance of each block of factors. The results are shown in Table 3. This consists of four models, with Model 1 including only personal factors as independent variables, Model 2 including both personal factors and organisational environmental factors, Model 3 including personal, organisational environmental factors and demographic factors, and Model 4 including chronic disease factors. The variances explained by each factor and each model were presented using Nagelkerke R square. The statistical significance level at *p* value less than 0.05 was used.

## 3. Results

### 3.1. Demographic Characteristics, Chronic Diseases and Depression

The final sample was 4,841 employees following exclusion of those who did not meet the inclusion criteria, and the removal of surveys with incomplete data. The mean ± SD age of the workers was 26.66 (5.65) years and the proportion of women was 79.3 per cent (see Table 1). There were significant differences between the depressed and non-depressed groups in age, gender, occupation, income and marital status (see Table 1). These variables as potential confounding factors were later entered into the logistic regression model and their influence on the relationship between individual and enterprise environment factors and depression was controlled.

**Table 1:** Depression by demographic variables n (%), personal factors and organisational environmental factors M(SD).

Variable	Total	Non-depressed (n = 3,234)	Depressed (n = 1,566)	χ^2^
**Age group**				
18–20	590	346 (58.6)	244 (41.4)	49.65 ^c^
21–30	3,044	2,019 (66.3)	1,025 (33.7)	
31–40	1,104	820 (74.3)	284 (25.7)	
>40	58	46 (79.3)	12 (20.7)	
**Sex**				
Male	991	688 (69.4)	303 (30.6)	2.39
Female	3,346	2,546 (66.8)	800 (33.2)	
**Occupation**				
CEO manager	183	150 (82.0)	33 (18.0)	44.47 ^c^
Business manager	376	286 (76.1)	90 (23.9)	
Counter director	782	523 (66.9)	259 (33.1)	
Officer	265	176 (66.4)	89 (33.6)	
Accounting record officer	553	342 (61.8)	211 (38.2))	
Excellent sales officers	422	300 (71.1)	122 (28.9)	
Sales officers	1,924	1,265 (65.7)	659 (34.3)	
Cashiers	166	106 (63.9)	60 (36.1)	
Technicians	129	86 (66.7)	43 (33.3)	
**Income group**				
3,000 and less	107	62 (57.9)	45 (42.1)	75.62 ^c^
3,000–5,999	197	120 (60.9)	77 (39.1)	
6,000–9,999	255	146 (57.3)	109 (42.7)	
10,000–14,999	398	235 (59.0)	163 (41.0)	
15,000–19,999	467	308 (66.0)	159 (34.0)	
20,000–29,999	821	518 (63.1)	303 (36.9)	
30,000–49,999	1,153	810 (70.3)	343 (29.7)	
50,000–59,999	581	411 (70.7)	170 (29.3)	
60,000–79,999	366	274 (74.9)	92 (25.1)	
>80,000	449	346 (77.1)	103 (22.9)	
**Job Type**				
Full time	4,746	3,199 (67.4)	1,547 (32.6)	0.83
Part time	34	24 (70.6)	10 (29.4)	
**Marital status**				
Not married	2,084	1,258 (60.4)	826 (39.6)	90.36 ^c^
Divorced	83	50 (60.2)	33 (39.8)	
Separated	41	26 (63.4)	15 (36.6)	
Married	2,586	1,896 (73.3)	690 (26.7)	
**Education**				
Primary school	19	14 (73.7)	5 (26.3)	2.59
Senior high school	2,573	1,719 (66.8)	854 (33.2)	
TAFE	250	170 (68.0)	80 (32.0)	
College	1,285	889 (69.2)	396 (30.8)	
Bachelor	324	221 (68.2)	103 (31.8)	
**Ability to meet work demands**				
Overall working ability M(SD)		8.17 (1.43)	7.35 (1.67)	17.63^ c^
Ability to meet physical demands M(SD)		4.31 (0.77)	3.94 (0.85)	16.28^ c^
Ability to meet psychological Demands M(SD)		4.18 (0.77)	3.69 (0.88)	25.72^ c^
**Resilience**		22.03 (2.99)	19.99 (3.22)	23.78^ c^
**Availability of health promotion program M(SD)**		44.67 (9.86)	42.57 (9.18)	7.11^ c^
**Enterprise ethos and culture M(SD)**		28.86 (3.39)	27.44 (3.49)	14.18^ c^
**Social environment M(SD)**		32.58 (4.21)	31.16 (4.15)	10.91^ c^
**Enterprise health policy M(SD)**		26.79 (4.16)	26.17 (4.12)	6.25^ c^

^c^ is *p* < 0.001. Chi-square test was used for categorical variable and t test was used for continuous variables.

As shown in Table 1, approximately 32.6 per cent of all enterprise workers reported GHQ scores of 10.5 or higher. There were significant differences between different age groups in the prevalence of depression: the younger 18–20 year old group had a higher prevalence of depression than other age groups (χ^2^ = 49.65, df = 3, *p* = 0.001). Regarding occupations, front line service people, including sales counter directors, officers, accounting record officers, sales officers and cashiers had a higher prevalence of depression than those in other occupations (χ^2^ = 44.47, df = 9, *p* = 0.001). Those at managerial level had the least prevalence of depression. Low-income earners with an annual income of less than 30,000 RMB (Chinese yuan) had a higher prevalence of depression than higher income groups (χ^2^ = 75.62, df = 4, *p* < 0.001). There were significant differences between different marital status groups as well. People who were unmarried, divorced or separated had a higher prevalence of depression than people who were married (χ^2^ = 90.36, df = 3, *p* < 0.001). There were no significant differences between men and women, and across education levels or job types. As there were significant differences between depression and non-depression group based on age, occupation, income and marital status, these factors as confounding factors were subsequently entered in the multiple logistic regression Model 3 where the relationship between depression and personal and organisational environmental factors was examined.

Employees reporting a chronic disease were significantly more likely than those in the normal and healthy group to have depression (Table 2). Having been told by a physician or nurse that one had anxiety, psychosis, schizophrenia, asthma or eating disorder was significantly associated with a greater risk of having depression, as was having had an injury. However, having had a type 2 diabetes, hypertension, cancer and heart attack was not related to be at risk of having depression. Variables including injury, asthma, anxiety, schizophrenia, psychosis, eating disorder, and other diseases will be entered into multiple logistic regression model (Model 4) to excluding the effect of chronic disease on the relationship between depression and individual and enterprise environmental factors.

**Table 2:** Association between depression and chronic disease factors.

	Total Number of participants	Non depress	Depress	χ^2^	*p*
		N (%)	N (%)		
**Injury**					
No	4,487	3,095 (69.0)	1,392 (31.0)	78.72	<0.001 ^c^
Yes	308	137 (44.5)	171 (55.5)		
**Diabetes**					
No	4,783	3,225 (67.4)	1,558 (32.6)	0.96	-
Yes	15	8 (53.3)	7 (46.7)		
**Type 2 Diabetes**					
No	4,786	3,225 (67.4)	1,561 (32.6)	-	-
Yes	12	8 (66.7)	7 (46.7)		
**Heart Disease**					
No	4,734	3,196 (67.5)	1,538 (32.5)	2.39	0.082
Yes	65	38 (58.5)	27 (41.5)		
**Hypertension**					
No	4,721	3,185 (67.5)	1,536 (32.5)	0.75	0.77
Yes	78	49 (62/8)	29 (37.2)		
**Cancer**					
No	4,791	3,229 (67.4)	1,562 (32.6)	0.09	0.56
Yes	8	5 (62.5)	3 (37.5)		
**Asthma**					
No	4,756	3,211 (67.5)	1,543 (32.4)	4.34	<0.05 ^a^
Yes	42	9 (29.0)	22 (71.0)		
**Anxiety**					
No	4,606	3,179 (69.0)	1,427 (31.0)	138.40	<0.001 ^c^
Yes	193	55 (28.5)	138 (71.5)		
**Schizophrenia **					
No	4,768	3,225 (67.6)	1,543 (32.4)	20.89	<0.001 ^c^
Yes	31	9 (29.0)	22 (71.0)		
**Psychosis**					
No	4,701	3,201 (68.1)	1,500 (31.9)	50.21	<0.001 ^c^
Yes	97	33 (34.0)	64 (66.0)		
**Eating disorders**					
No	4,059	2,842 (70.0)	1,217 (30.0)	81.71	<0.001 ^c^
Yes	737	391 (53.1)	346 (46.9)		
**Other diseases**					
No	4,309	2,948 (68.4)	1,361 (31.6)	21.76	0.001 ^c^
Yes	485	281 (57.9)	204 (42.1)		

Chi square analysis was used for the table. Statistical significance: ^a^ is p < 0.05; ^c^ is p < 0.001.

### 3.2. Depressive Symptoms and Employees’ Ability to Meet Work Demands

As shown in Table 3, employees’ whose GHQ score was 10.5 or greater were defined as having current depression. Table 1 also shows that the resilience score of depressed groups, who had higher GHQ scores and perceptions of lower overall working ability and lower ability to meet the physical and mental demands of work, was lower than the non-depressed group. The overall low level of work ability, low level of ability to meet the physical and mental demands of work, and low resilience scores were significantly related to an increased chance of depression. The association of their overall low level of working ability, ability to meet the physical and mental demands of work, and low level of resilience to depression is statistically significant, explaining 18.7 percent of the variance (see Model 1). The association between the personal level factors and depression is significant even when organisational environmental factors (Model 2), demographic factors (Model 3) and chronic disease factors (Model 4) are included in the regression model. Organisational environmental, demographic and chronic disease factors only explained 0.5%, 1.8% and 1.7% respectively of the variance in the prediction of depression.

**Table 3:** Association between depression and individual work ability and enterprise environment factors.

	Model 1	Model 2	Model 3	Model 4
	OR (95% CI)	OR (95% CI)	OR (95% CI)	
**Ability to meet work demands**				
Overall working ability	0.89 (0.84 to 0.93) ^c^	0.89 (0.85 to 0.94) ^c^	0.86 (0.82 to 0.92) ^c^	0.91 (0.87 to 0.95) ^c^
Ability to meet physical demands	0.80 (0.74 to 0.87) ^c^	0.81 (0.75 to 0.89) ^c^	0.79 (0.71 to 0.88) ^c^	0.83 (0.77 to 0.90) ^c^
Ability to meet psycho demands	0.59 (0.54 to 0.64) ^c^	0.60 (0.55 to 0.66) ^c^	0.63 (0.56 to 0.70) ^c^	0.62 (0.57 to 0.68) ^c^
**Resilience**	0.86 (0.84 to 0.88) ^c^	0.87 (0.85 to 0.89) ^c^	0.83 (0.80 to 0.89) ^c^	0.87 (0.85 to 0.89) ^c^
**Availability of Health promotion programs**		1.00 (0.99 to 1.01)	0.99 (0.98 to 1.01)	1.00 (0.99 to 1.01)
**Enterprise ethos and culture**		0.96 (0.94 to 0.98) ^c^	0.92 (0.91 to 0.95) ^c^	0.96 (0.94 to 0.98) ^c^
**Social environment**		0.99 (0.91 to 1.01)	0.95 (0.94 to 1.01)	0.99 (0.98 to 1.02)
**Enterprise health policy**		0.98 (0.95 to 1.01)	0.97 (0.95 to 1.02)	1.00 (0.99 to 1.02)
**Occupation**				
CEOs			1	1
Business managers			1.12 (0.80 to 1.57)	1.13 (0.81 to 1.59)
Counter managers			1.09 (0.79 to 1.51)	1.15 (0.82 to 1.60)
Officers			1.12 (0.77 to 1.63)	1.17 (0.80 to 1.72)
Accounting record officers			0.95 (0.57 to 1.03)	1.03 (0.72 to 1.47)
Excellent sales officers			0.73 (0.51 to 1.03)	0.79 (0.55 to 1.12)
Sales guiding officers			0.79 (0.56 to 1.09)	0.86 (0.62 to 1.19)
Cashiers			0.79 (0.51 to 1.22)	0.85 (0.54 to 1.33)
Technicians			0.94 (0.60 to 1.47)	1.05 (0.67 to 1.64)
**Income**				
More than RMB 80,000			1	1
3,000 RMB			1.70 (1.16 to 3.74) ^a^	1.67 (1.08 to 2.59) ^a^
3,000–5,999			1.47 (0.87 to 2.35) ^a^	1.49 (1.05) to 2.11) ^a^
6,000–9,999			1.49 (0.99 to 2.51) ^a^	1.56 (1.12 to 2.16) ^a^
10,000–14,999			1.38 (1.07 to 2.46) ^a^	1.36 (1.02 to 1.81) ^a^
15,000–19,999			1.27 (.81 to 1.87)	1.34 (1.01 to 1.76) ^a^
20,000–29,000			1.43 (1.10 to 2.34) ^b^	1.46 (1.14 to 1.86) ^b^
30,000–39,000			1.26 (0.88 to 1.82)	1.18 (0.94 to 1.48)
40,000–59,999			1.01 (0.67 to 1.53)	1.25 (0.98 to 1.60)
60,000–79,999			1.40 (0.89 to 2.20)	1.09 (0.84 ot 1.42)
**Age**				
18–20			1.49 (0.86 to 2.61)	1.38 (0.79 to 2.40)
21–30			1.43 (0.86 to 2.40)	1.30 (0.77 to 2.17)
31–40			1.58 (0.94 to 2.64)	1.49 (0.89 to 2.43)
<40			1	1
**Marital status**				
Married			1	1
Not married			1.57 (1.08 to 2.31) ^b^	1.25 (1.08 to 1.45) ^b^
Divorced			3.38 (1.47 to 7.79) ^b^	1.59 (1.04 to 2.43) ^a^
Separated			2.55 (0.73 to 8.92)	1.13 (0.61 to 2.07)
**Injury**				1.47 (1.16 to 1.86) ^c^
**Asthma**				0.91 (0.48 to 1.72)
**Anxiety**				3.06 (2.24 to 4.18) ^c^
**Schizerfrania**				1.35 (0.58 to 3.08)
**Other psychosis**				1.17 (0.75 to 1.83)
**Eating disorder**				1.41 (1.20 to 1.66) ^c^
**Other diseases**				1.15 (0.95 to 1.39)
**Total variances explained**	18.7%	19.2%	21.0%	22.7%

Multiple logistic regression was used in the four models. Statistical significance: ^a^ is *p* < 0.05; ^b^ is *p* < 0.01; ^c^ is *p* < 0.001.

### 3.3. Depressive Symptoms and Organisational Environmental Factors

The data from the 13 enterprises were combined in a logistic regression analysis to test the association between current depression with organisational environment factors, including ethos and culture, social environment, availability of health promotion program, and enterprise health policies. These factors are listed as independent variables in the logistic regression model in Table 3. Potential confounders (age, occupation, income and marital status) (see Model 3 in Table 3) and chronic disease factors (see Model 4 in Table 3) were entered into the model and controlled. The results show a significant association between depression and the organisational environmental factors, suggesting that enterprise ethos and culture, are inversely and significantly related to current depression. High level of enterprise ethos and culture factors, including opportunity to encourage employees’ success, appropriate level of performance expectations, respect and recognition of employees’ achievement, are significantly related to less probability to have depression.

### 3.4. Depression and Demographic Factors and Chronic Disease

As can be seen in Table 3, income, occupation and job type are significantly related to depression. The prevalence of depression was greater for those earning an income less than 30,000 RMB compared to those earning more than RMB80,000, those who are single, separated or divorced compared to those who are married, men compared to women, and clerical officers or service workers compared to managers or executives. Significantly lower depression rates occurred for those aged 40 years and older compared with those aged 18–20 years, married people compared with single or unmarried people, and front line workers compared with managers or executives. Employees reporting a chronic disease were significantly more likely than those in the normal and healthy group to have depression (Table 3). Having been told by a physician or nurse that one had anxiety, psychosis, schizophrenia, asthma or an eating disorder was significantly associated with a greater risk of having depression, as was having had an injury.

In summary, all personal factors including inability to meet physical and psychological work demand, and low level of resilience are significantly related to depression and explained the largest variances in the prediction of depression models, whereas only one organizational factor (enterprise ethos and culture) is negatively related to depression. Therefore, the research hypothesis is partially supported.

## 4. Discussion

Participants in this study represent a diverse group of employees in the private retail sector in terms of demographic characteristics and occupational types, and are therefore fairly typical of workers in this industry in China. The proportion of employees who had cut off score more than 10.5 was 32.6 per cent as measured by GHQ-30. A score of 10.5 or higher on the GHQ-30 had a 25.0 % positive predictive value for the detection of true major depression in this study. Therefore, 8.15% of enterprise employees would have experienced major depression, a figure is comparable to that in a national study of mental disorders reported by Kessler and colleagues [24]. In addition, 24.45% of participants had elevated stress is also a big concern considering the link between stress in the workplace and increased risk of disease and ill-health [25,26].

The most commonly identified issue contributing to depression and stress among participants was their inability to meet physical and mental work demands, a finding consistent with previous studies [27]. With rapid economic development in China, privately owned enterprises are facing great pressure to compete and survive in the market, inevitably leading to increased productivity demands of employees.

Not surprisingly, 80 per cent of participants strongly agreed that organisational expectations regarding performance were high, resulting in unmanageable stress levels for those employees unable to meet the physical and psychological demands of their jobs.

An imbalance between a low ability to control the job and high demand from managers was related to depression and stress in our study. This result supports the job demand-control model, which suggests that individuals experience role strain and subsequently negative health effects when the demands of the job exceed their perceived control. In short, higher demands lead to higher strain [4], and low control and low work ability to meet job demands can lead to depression and stress.

Consistent with previous research, resilience in dealing with stress and job challenges, and ability to cope with adversity is an important factor in adapting and adjusting to change and challenging situations. Such traits assist people to manage stressful situations and therefore avoid negative outcomes such as depression and stress [28]. Our findings provide evidence that Chinese employees who had a high level of resilience had a reduced probability of having depression and stress. The results support the proposition that an employee’s ability to bounce back from stressful events plays an important role in the workplace to buffer the adverse effect of job stress and maintain a balance between work ability and job demands [28].

Negative perceptions of the enterprise’s ethos and culture were also significantly related to an increased likelihood of having depression and distress, a result that is also consistent with studies from Western countries [8,15,29,30]. Our findings support the culture-work-health model, which focuses on the organisation as a whole and sees a positive work culture, including worker autonomy, good job design and provision of social support, as a key buffer to depression. In our study, cultural issues, including the way employees are respected, recognised and valued, and positive relationships between managers and workers, was related to a reduced probability of having depression and stress.

Consistent with previous studies, health problems, particularly chronic conditions such as injury, asthma, and other psychiatric conditions were associated with greater likelihood of depression and stress, as was having had an injury [31,32,33]. People with chronic conditions and injuries often have less opportunity to participate in health promotion activities in the enterprise environment, thereby reducing their opportunity to manage their condition, improve and their sense of self-efficacy and therefore avoid depression and stress.

Depression and stress was greater among those who were divorced or unmarried. Further, a higher percentage of low income earners (less than 30,000 RMB) were more likely to have symptoms of depression than those with incomes over 80,000 RMB. Previous research has associated major changes in marital status and related financial stress, work/life imbalance and life dissatisfaction with higher levels of depression. We also found that employees who are frontline staff, such as sales officers, counter managers and accounting record officers had the highest depression scores. To some extent this may be due to the frequent stressful interactions with hostile and difficult customers associated with these roles.

The study results suggest the following implications. First, our results emphasise the need for managers to provide mental health promotion activities and improve employees’ ability to meet physical and psychological demand of the work in order to help reduce the probability of depression and stress among employees in these occupations [16]. Second, it is important to develop and provide supportive environments, the creation of a supportive ethos and enterprise culture is fundamental to health promotion efforts, particularly given the strong association between depression and stress, and support for social and mental health, especially for those front line, blue collar and low income level employees, and people with chronic condition.

The primary limitation of the study concerned the nature of the sample. It was only conducted with non-state-owned enterprises, and only in two provinces, thus, caution needs to be taken in generalizing the results and applying them to other provinces and state-owned enterprises. In addition, GHQ-30 is a self-administered screening instrument designed to detect depression, and is used in surveys to identify potential cases, leaving the task of diagnosing actual disorder to a clinical or psychiatric interview [19]. Due to funding limitations, we were not able to conduct clinical interviews with every employee who had GHQ-30 score 10.5 and above and consequently this study cannot differentiate participants who fulfilled the Diagnostic and Statistical Manual of Mental Disorders or other diagnostic criteria for major depression and people who were in elevated level of stress. Despite these limitations, this was the first study in China to focus on factors which may be related to depression and stress, and work productivity in privately owned enterprises in China. Future studies should apply clinical interview techniques to increase the accuracy of diagnoses of depression in the workplace. Data should also be collected from state-owned enterprises, as well as private enterprises, to determine the generalizability of findings across industry sectors.

## 5. Conclusions

The findings of the study suggests that an intervention program targeting individual capacity to meet the physical and psychological demands of work, promote sense of resilience, and improve workplace ethos and culture factors is needed to reduce the feelings of depression and stress in the workplace. Providing counselling, health and wellness services to manage depression and stress may be an effective approach to lowering the occurrence of this condition in the workplace.
